# Transcranial Alternating Current Stimulation for Pain: Mixed Evidence and the Path to Precision Neuromodulation

**DOI:** 10.3390/brainsci16020152

**Published:** 2026-01-29

**Authors:** Yaser Fathi, Amin Dehghani, David M. Gantz, Giulia Liberati, Tor D. Wager

**Affiliations:** 1 Institute of Neuroscience, Université Catholique de Louvain, 1200 Brussels, Belgium; yaser.fathiarateh@uclouvain.be (Y.F.); giulia.liberati@uclouvain.be (G.L.); 2 Department of Psychological and Brain Sciences, Dartmouth College, Hanover, NH 03755, USA; amin.dehghani@dartmouth.edu (A.D.);; 3 Psychological Sciences Research Institute, Université Catholique de Louvain, 1348 Louvain-la-Neuve, Belgium

**Keywords:** tACS, pain perception, neural oscillations, alpha power, experimental pain, chronic pain, brain imaging, electrophysiological recordings

## Abstract

Neural oscillations are fundamental to the integration of sensory, affective, and cognitive processes that contribute to pain perception. Transcranial alternating current stimulation (tACS) provides a valuable tool for investigating and modulating these oscillatory dynamics. In this review, we examine the effects of tACS on pain perception and pain-related oscillations in both healthy participants and individuals with chronic pain, highlighting methodological variability and mechanistic uncertainties that may contribute to mixed findings. We identified 14 studies, including 9 studies of experimental pain in healthy individuals and 5 of clinical pain disorders, comparing tACS to sham. Somatosensory alpha was the most frequently targeted oscillatory feature. Results varied considerably. Several studies reported reductions in pain, increases in alpha power, or changes in sensorimotor and prefrontal connectivity, but others showed no meaningful neural or behavioral effects. Out of the 14 studies, 6 demonstrated analgesic benefits and 2 showed improvements only under specific conditions or within subgroups, for a total of 8/14 studies with positive findings. Possible sources of heterogeneity include variation in stimulation duration, electrode montage, frequency alignment with individual rhythms, contextual state, and anatomical and neurophysiological differences across individuals. Pre-registered studies with sufficient power are needed to replicate effects within the most promising intervention protocols to establish a foundation in the field. We also recommend inclusion of brain imaging or electrophysiological recordings to verify whether stimulation effectively modulates the targeted neural oscillations. Finally, recent methodological advances, including phase-specific tACS, amplitude-modulated tACS, and individualized electric-field modeling, offer new opportunities to enhance mechanistic precision and clinical applicability. We argue that by integrating these approaches, future research can move beyond fixed, one-size-fits-all protocols toward personalized, state-dependent, closed-loop tACS approaches. Exploring these frontiers will transform tACS from an exploratory tool into a reliable intervention for pain.

## 1. Introduction

Neural oscillations reflect neurophysiological mechanisms that support the integration of sensory, affective and cognitive processes involved in pain perception [1]. Human rhythms have historically been characterized by their frequency, with major frequency bands including (from low to high) delta (0.5–4 Hz), theta (4–7 Hz), alpha (7–12 Hz), beta (12–30 Hz), and gamma (30–55 Hz for low gamma and 55–200 for high gamma). They are measured using time-frequency decomposition of scalp electroencephalography (EEG) or, less frequently, intracranial EEG (iEEG). These rhythms reflect synchrony across populations of neurons and are thought to play a fundamental role in regulating which neurons are excitable at specific times, allowing the formation of integrated networks of neuronal subpopulations across the brain [2]. Alpha rhythms are found across the brain and are associated with multiple functions, including attention [3] and sensory gating, where higher alpha power is thought to reflect stronger inhibition of incoming sensory input and lower cortical excitability—providing an important framework for interpreting alpha changes in pain [4]. Alpha 1 power (7–10 Hz), measured over temporal and somatosensory regions, has been found to negatively correlate with pain ratings both at rest and during phasic thermal stimulation [5]. Alpha amplitude is also shaped by cognitive and contextual factors, decreasing with attention to pain [6] and increasing with the expectation of pain relief [7]. A slowing of the individual peak alpha frequency (PAF)—reported in chronic pain conditions [8,9,10,11,12,13]—often occurs with increased theta and gamma activity, forming a pattern known as thalamocortical dysrhythmia, which is thought to reflect altered thalamic bursting and cortical hyperexcitability. In healthy individuals, lower PAF has been associated with increased pain sensitivity [14] and reduced nociceptive facilitation [15].

These associations offer promise for understanding pain and provide targets for neuromodulatory interventions. However, there is still uncertainty about how consistently brain oscillations relate to pain. Recent large-scale studies report no significant relationship between resting PAF and pain sensitivity, supporting the conclusion that no EEG biomarker, including alpha slowing, has yet been firmly established as a reliable or generalizable marker of chronic pain [16,17]. Instead, alpha dynamics appear to reflect broader cross-frequency reorganization within cortical networks. For example, Bott et al. [18] demonstrated that expectations modulate prefrontal to somatosensory connectivity at alpha frequencies, whereas gamma band coupling encodes prediction errors, consistent with predictive coding models of pain. In clinical populations, frequency-specific patterns further highlight distributed network involvement. In chronic low back pain, theta and alpha activity in the orbitofrontal cortex were linked to localized hypersensitivity, while anterior cingulate gamma and dorsolateral prefrontal theta tracked generalized hyperalgesia [19]. In neuropathic pain, higher alpha and lower delta and theta power were associated with increased pain intensity [20]. Thus, a growing body of evidence is at odds with the simple characterization of pain as a high-theta, low-alpha phenomenon. The source locations of measured rhythms, individual differences, and pain types and states may matter in complex ways. Human iEEG work further underscores this heterogeneity in oscillatory correlates of pain across individuals [21], though there may be some consistency across individuals as well. In Shirvalkar et al.’s study [21], which involved sustained iEEG recording in individuals with chronic pain across ~120 days, the most consistent correlates of pain were decreased anterior cingulate alpha/beta power for experimental heat pain and reduced orbitofrontal delta for chronic spontaneous pain.

Given the importance of brain rhythms in circuit integration and behavior, including pain, transcranial alternating current stimulation (tACS) provides a relatively unique and important opportunity to stimulate the brain at particular frequencies [22]. tACS is a noninvasive method that delivers low-intensity sinusoidal electrical currents through scalp electrodes. When tACS is applied at a frequency close to the brain’s own ongoing rhythm, it can entrain neuronal activity by aligning the phase of neural oscillations with the stimulation waveform [23]. This effect is believed to follow the so-called Arnold tongue principle, in which the strength of entrainment increases as the stimulation frequency more closely matches the endogenous oscillatory frequency [24,25]. Another proposed mechanism involves the facilitation of synaptic plasticity via spike-timing-dependent plasticity (STDP), which may allow tACS to enhance oscillatory power at the stimulated frequency [26]. The application of tACS at the PAF has been proposed to promote effective neuronal synchronization [27], to stabilize PAF against potential slowing [28], and to augment its power and gating functions. More broadly, tACS at frequencies beyond alpha may be warranted, as pain-related cortical networks show complex cross-frequency reorganizations rather than isolated alpha-band alterations [18,19,20,21].

## 2. Methods (Narrative Literature Approach)

Literature was identified through iterative searches in PubMed using combinations of keywords including “tACS”, “transcranial alternating current stimulation”, “pain”, “chronic pain”, “experimental pain”, “EEG”, and “neural oscillations”. Additional articles were identified from the reference lists of the analyzed articles. Searches focused on peer-reviewed studies in the literature published up to November 2025. We included randomized, controlled studies examining the impact of tACS on either experimentally induced pain outcomes in healthy adults or clinical pain outcomes in chronic pain patient groups, relative to a control condition. Control conditions included either sham stimulation or an alternative active comparator transcranial electrical stimulation (tES) condition.

## 3. Results: Current Evidence on the Effects of tACS on Experimental and Clinical Pain

Across the growing literature on tACS, studies have examined whether rhythmic electrical stimulation can alter pain perception, modulate oscillatory activity, or reduce chronic pain symptoms. Although findings are heterogeneous, converging evidence indicates that tACS can modulate pain perception and pain-related neural activity under specific contextual and stimulation conditions. Specifically, outcome variability appears to depend on stimulation parameters (frequency, montage, intensity, duration), individual neurophysiology, and task.

Table 1 summarizes the key design features and outcomes across all published tACS experimental and clinical pain studies included in this review.

These studies exhibit substantial methodological variability. Sample sizes are typically small, and stimulation parameters, including frequency, intensity, montage, electrode configuration, and duration, vary widely. Power is also likely to be an issue. To detect a moderate effect size of Cohen’s *d* = 0.5 with 80% power, N = 34 participants are required for a within-person crossover, and N = 130 (65 per group) are required for a between-person comparison across groups. By this standard, only two studies in Table 1 are adequately powered to detect moderate effect sizes.

### 3.1. Evidence from Experimental Pain Studies

Alpha-frequency stimulation over the somatosensory cortex has shown mixed results. Arendsen et al. [29] reported a reduction in both pain intensity and unpleasantness during α-tACS, but only under conditions of uncertain pain expectation, suggesting an interaction between oscillatory modulation and cognitive context. In contrast, May et al. [32] found no modulation of pain or autonomic responses during α- or γ-tACS over the prefrontal cortex or S1, with Bayesian analyses supporting the null hypothesis for most conditions. Some studies targeting SM1 with α-tACS also showed dissociations between neural and behavioral effects. Peng et al. [33] reported reduced pain-evoked activity and decreased SM1-network connectivity, although pain ratings did not significantly differ from sham. Fathi et al. [28], using individualized somatosensory PAF stimulation, likewise observed no overall analgesic effect, although exploratory analyses suggested reduced sensitization in women and possible stabilization of both alpha rhythms and pain perception. Li et al. [31] reported no reduction in pain but observed disruption of laser-evoked potential habituation and changes in α_1_ and θ activity during anticipation. Mediation analyses further suggested competing influences on pain, with enhanced α_1_ oscillations tending toward analgesia, whereas increased θ activity was associated with hyperalgesia.

Some studies have shown more robust perceptual effects using longer stimulation durations and high-definition (HD) montages. Qi et al. [34] reported that 10 Hz HD-tACS over SM1 reduced capsaicin-induced pain, and resting α power and pain-related θ modulation predicted individual responsiveness. Sun et al. [35] found that HD-tACS over the dorsolateral prefrontal cortex (DLPFC) reduced pain and produced delayed increases in low γ power, although EEG was recorded only at rest.

Other frequency bands have also been explored. Ikarashi et al. [30] demonstrated that θ- and β-tACS over the DLPFC increased heat pain thresholds, with θ-tACS showing an inverted U-shaped relationship between modeled electric field strength and analgesic efficacy, suggesting that both insufficient and excessive stimulation can reduce efficacy.

Finally, interpersonal and affective factors can shape tACS outcomes. Takeuchi and Terui [36] showed that synchronous dual-brain α-tACS enhanced social touch-induced analgesia in low-empathy individuals, even though overall pain ratings did not differ across conditions. These results highlight that tACS effects may depend not only on stimulation parameters but also on social and emotional context.

Together, findings on experimental pain show that tACS can alter pain-related neural activity and occasionally reduce pain perception, but results vary considerably across studies. More consistent effects may emerge when stimulation is longer, spatially focused, tailored to individual oscillatory properties, or applied in contexts that shape pain processing, such as expectation, uncertainty or social interaction.

### 3.2. Evidence from Chronic Pain Studies

Clinical studies generally provide more consistent indications that tACS can influence ongoing chronic pain symptoms, although sample sizes are small and protocols vary widely.

In chronic low back pain, Ahn et al. [37] found that 10 Hz bifrontal tACS reduced pain ratings relative to sham and increased somatosensory α power, with greater α enhancement predicting larger reductions in pain and disability. Prim et al. [39] also applied 10 Hz tACS over prefrontal regions and observed autonomic modulation (increased heart rate variability) and a higher proportion of clinical responders in the active condition, although group-level pain reductions did not reach statistical significance.

Fibromyalgia studies provide mixed but informative results. Bernardi et al. [39] tailored the frequency of stimulation using 4 Hz for slow-rhythm abnormalities and 30 Hz for fast-rhythm abnormalities and observed reductions in pain intensity, improvements in cognitive and affective symptoms, and increases in α_1_ power, with effects diminishing after four weeks. In contrast, Lin et al. [40] applied 50 Hz HD-tACS over M1 and found no significant differences between active and sham overall, although active stimulation reduced fibromyalgia impact scores relative to baseline.

Clinical protocols also vary widely in stimulation frequency, intensity, montage, electrode configuration, and number of sessions, ranging from single-session interventions to multi-day treatment protocols. Despite this heterogeneity, several patterns emerge. Longer or repeated stimulation tends to show more consistent symptom improvement. Tailoring stimulation frequency based on patient stratification may yield stronger effects than using fixed frequencies. While increases in α power following bifrontal stimulation and normalization of abnormal oscillatory patterns following M1 stimulation have been associated with reductions in pain, further studies are required to confirm the robustness and generalizability of these associations.

## 4. Discussion: Sources of Inconsistencies, Limitations, and Going Beyond Conventional tACS

Uncertainty about the robustness and mechanisms of tES effects is not unique to pain research and has been emphasized in recent general reviews of the field. Bland and Sale [42] highlighted several fundamental challenges for tACS, including weak cortical currents, limitations in concurrent neuroimaging due to stimulation artifacts, and the difficulty of dissociating genuine neural entrainment from indirect or peripheral effects. Potential methodological solutions have also been discussed. Here, we focus on how these general challenges extend to pain research and consider recent developments in the field that may help improve mechanistic clarity and reproducibility.

### 4.1. Mechanistic Uncertainty Driven by Target and Frequency Variability

Pain processing is not localized in a single brain region, but it is highly distributed and involves contributions from sensorimotor, prefrontal, limbic, and cingulo-opercular circuits, each oscillating at multiple characteristic rhythms [1]. Moreover, individuals suffering from chronic pain can also exhibit elevated activity in limbic brain regions [43,44,45] and other brain network-level abnormalities [46,47,48,49,50], further complicating the search for analgesic targets and frequencies for clinical applications.

Although alpha activity prior to pain onset correlates with lower pain ratings in some studies [5,51,52], somatosensory alpha-tACS has not consistently modulated pain. For instance, rigorous sham-controlled studies of tonic pain failed to show robust analgesic effects for either somatosensory alpha- or prefrontal gamma-tACS, suggesting that simple one-to-one mappings between rhythm and perception may be insufficient [32]. Power is likely an issue, however, as mentioned above, and larger pre-registered studies must be conducted to more definitively address this issue. Complementary imaging-based work has demonstrated that alpha-tACS over M1 reduces both bilateral SM1 activity and connectivity with pain-related regions, with changes emerging most prominently after stimulation rather than during, pointing toward plasticity mechanisms that extend beyond immediate entrainment [33].

The mechanistic uncertainties surrounding target site selection and frequency tuning therefore represent a critical barrier: without biomarker-guided strategies, stimulation protocols risk inconsistency, with clinical trials reporting modest or null effects despite promising preclinical rationales [53]. Establishing causal links between frequency-specific entrainment, network-level modulation, and pain reduction is therefore essential for moving toward precision neuromodulation.

### 4.2. Sources of Inter-Individual Variability

A major challenge for effective tACS interventions is the substantial inter-individual variability in stimulation outcomes. This variability arises from a combination of anatomical, physiological, and contextual factors that shape the degree to which electrical fields engage targeted neural circuits. Structural MRI-informed modeling suggests that individual differences in head and brain anatomy lead to substantial variability in intracranial electric field strength and distribution, even when identical stimulation montages are used [30]. As a result, two individuals receiving the same nominal current may experience very different levels of cortical engagement, and behavioral efficacy may follow non-linear patterns, including inverted U relationships between field magnitude and analgesic effects. Such effects, however, remain to be replicated in well-powered studies.

Physiological traits further contribute to variability. Baseline oscillatory dynamics, such as resting alpha power and PAF, are thought to be predictors of pain sensitivity and of responsiveness to stimulation. In controlled experiments, resting alpha power and pain-related theta reactivity jointly mediated responses to alpha HD-tACS over SM1.

Sex and gender differences may also be a factor. Complementary work has shown sex-specific effects in which women exhibited stronger modulation of heat pain thresholds [28]. Biological rhythms, including the menstrual cycle, introduce additional variability through their effects on pain thresholds, cortical excitability, and oscillatory activity.

### 4.3. Outcomes Measures

Variability in outcome measures and pain models further limits comparability across studies. Experimental paradigms include tonic heat [32], laser stimulation [31,33], electrical pain [36], pressure pain [29], and capsaicin-induced sustained pain [34,35], each engaging partially distinct sensory and cognitive processes. Chronic pain studies involve different diagnoses, symptom profiles, and comorbid conditions. Neural readouts also vary widely, with some studies relying solely on behavioral ratings [29], others measuring resting EEG before and after stimulation [28,32], and still others collecting fMRI activity during pain [33]. Differences in the timing of measurements relative to stimulation further add to inconsistency. Together, these factors make it difficult to identify stimulation features that consistently influence pain or pain-related neural activity. Improved comparability could be achieved by adopting more standardized pain models and outcome measures, clearly distinguishing experimental from clinical pain contexts, and systematically aligning neural readouts and assessment time points with hypothesized mechanisms. Such methodological convergence would facilitate cross-study comparisons, meta-analytic synthesis, and a more coherent understanding of how stimulation parameters relate to pain and pain-related neural activity.

### 4.4. Blinding Challenges

Blinding quality is another major concern, as sensory experiences during stimulation can differ substantially between active and sham conditions. Because expectation strongly modulates pain perception, inconsistent sham procedures introduce an important confound that may obscure or exaggerate stimulation effects. Across the studies summarized in Table 1, only a limited subset of those reporting significant neural or analgesic effects also demonstrated adequate blinding as verified by formal assessment. Adequate blinding was typically defined by post-session questionnaires assessing participants’ ability to guess stimulation condition, with chance-level accuracy indicating successful blinding. Such assessments were explicitly reported in several double-blind studies, including May et al. [32], Fathi et al. [28], Qi et al. [34], Peng et al. [33], Ahn et al. [37], and Prim et al. [41], all of which employed ramp-up and ramp-down sham protocols designed to mimic initial sensory experiences. Among these, some studies reported clear neural modulation without robust behavioral analgesia [33], whereas others showed pain reduction alongside verified blinding [34,37]. In contrast, multiple studies reporting significant reductions in pain intensity or thresholds did not report formal blinding assessments or relied on single-blind or between-subject designs, including Arendsen et al. [29], Ikarashi et al. [30], Sun et al. [35], and Bernardi et al. [39]. Clinical studies involving longer or repeated stimulation sessions also appear especially vulnerable to partial unblinding, as sensory differences may accumulate over time [37,39,41]. Overall, while several studies in Table 1 report significant neural or analgesic effects, only a small number combine these findings with empirically verified participant blinding, highlighting blinding quality as a critical and inconsistently addressed source of heterogeneity in the current tACS pain literature.

In short, across the 14 sham-controlled tACS pain studies summarized in Table 1, only 6 clearly reported a participant blinding assessment, highlighting the need for more systematic evaluation and transparent reporting of blinding quality in tACS pain research. Blinding in tACS is inherently challenging because cutaneous sensations (tingling, itching, warmth, phosphenes) can differ between active and sham conditions, particularly at higher intensities. While a trade-off between maintaining blinding and ensuring effective tACS application has been discussed in [32], recent unconventional tACS approaches using high-carrier frequencies modulated at lower frequencies (see Section 4.5.1) suggest that higher intensities can be applied while preserving successful blinding [54]. We further recommend the use of an “expectation-neutral blinding assessment,” defined as allocation-belief questions designed to minimize demand characteristics, for example, by allowing “unsure” responses and confidence ratings rather than implying that active and sham conditions must feel different. This approach is motivated by evidence that participant beliefs can influence subjective outcomes [55] and that traditional end-of-study condition guesses may poorly reflect true sensory detectability [56]. Importantly, such blinding procedures should be pilot tested to verify blinding success before full-scale experiments (see [57] for an example).

### 4.5. Beyond Conventional tACS

Here, we review recent developments in unconventional tACS techniques and discuss their potential to address the inconsistencies and limitations outlined above.

#### 4.5.1. Amplitude-Modulated tACS (AM-tACS)

While many studies focus on aftereffects linked to plasticity, the online effects of tACS—particularly neuronal entrainment during stimulation—are less understood. Directly tracking neural activity during tACS would help clarify these mechanisms, but concurrent EEG recordings are technically challenging due to strong stimulation artifacts. Beyond this, current tACS protocols are typically fixed throughout stimulation, whether individualized or not, whereas optimal neuromodulation likely requires parameters that adapt dynamically to ongoing brain activity. Real-time monitoring would enable such adaptive or closed-loop frameworks by aligning stimulation parameters to moment-to-moment oscillatory dynamics.

**Figure 1:**
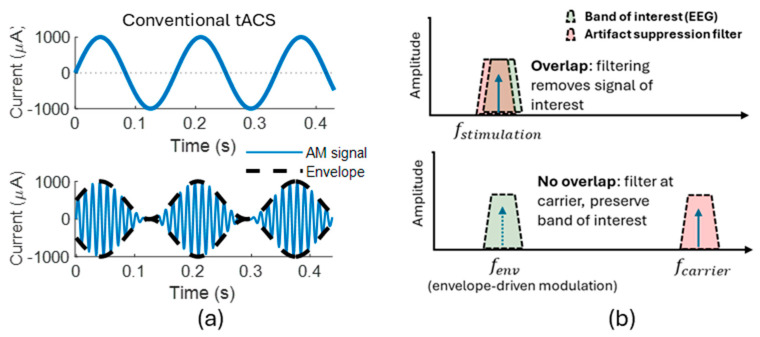
(**a**) AM-tACS delivers a high-frequency carrier whose amplitude is modulated at a lower envelope frequency. (**b**) During EEG recording, stimulation artifacts mainly cluster around the carrier frequency. Because of this spectral separation, filtering can attenuate the carrier-related artifacts while preserving the low-frequency envelope content. The arrows indicate the main frequency components of the stimulation signal.

To overcome stimulation artifacts and enable online monitoring of neuronal entrainment, Witkowski et al. [58] introduced amplitude-modulated tACS (AM-tACS), where the intended low-frequency stimulation pattern is embedded within a high-frequency carrier signal. The high-frequency carrier is set sufficiently high to avoid phase locking of neuronal activity to the carrier. For recordings, applying an appropriate low-pass filter removes the high-frequency components of the stimulation signal. (Figure 1). However, nonlinearities in stimulation and recording hardware can introduce residual artifacts, and additional denoising approaches have been developed to more reliably recover the neural activity [54,59,60].

While AM-tACS provides a valuable tool for artifact-free recording, its efficacy as a neuromodulatory technique relative to conventional tACS requires further validation [61].

#### 4.5.2. Phase-Dependent and State-Dependent Modulation in tACS

While tACS is often assumed to entrain brain oscillations at their endogenous frequency, recent evidence indicates that it interacts with ongoing neural activity rather than merely amplifying it. Using electrophysiological recordings in awake rhesus monkeys, Krause et al. [62] demonstrated that tACS does not simply impose rhythmicity on neurons but instead competes with intrinsic oscillations for control of spike timing. These recordings revealed that neurons with weak baseline entrainment became more rhythmic during tACS, whereas strongly entrained neurons often lost synchrony. The findings explain variability in human tACS/EEG studies and suggest opportunities for both strengthening and desynchronizing neural activity.

In humans, Fiene et al. [63] provided complementary evidence by pairing visual flicker with occipital tACS at different phase shifts. They showed that steady-state visual responses were modulated in a phase-dependent manner and that the optimal phase shift correlated with each participant’s cortical response delay. These findings highlight that the effectiveness of tACS depends critically on the timing of stimulation relative to ongoing brain rhythms. Haslacher et al. [54,60] provided evidence that the impact of tACS critically depends on its phase relationship to ongoing oscillations. They showed that stimulation at specific phases could enhance or suppress both neural activity and perceptual dominance and later demonstrated that working memory performance was maximized at distinct phase lags that aligned with changes in frontoparietal alpha synchrony. Another example showing the importance of timing is provided by Schreglmann et al. [64], who demonstrated that non-invasive cerebellar tACS phase-locked to the tremor rhythm in essential tremor patients suppressed tremor amplitude by disrupting the temporal coherence of pathological oscillations, with the effective phase lag varying across individuals.

Together, these studies demonstrate that the efficacy and even the direction of tACS effects (enhancement or suppression) are not fixed but depend on phase alignment with ongoing neural activity and brain state, varying not only across individuals but also across time within the same individual. For pain modulation, this implies that the analgesic effect of, for example, alpha-tACS may depend on applying the stimulation at the correct phase of the individual’s ongoing sensorimotor alpha rhythm. Current protocols do not account for this, potentially explaining why some participants respond and others do not. At the same time, state-dependent suppression can be therapeutically exploited. For instance, in neuropathic pain conditions characterized by thalamocortical dysrhythmia, tACS could be tuned to selectively suppress aberrant low-frequency theta or delta oscillations.

#### 4.5.3. Temporal Interference Stimulation (TIS)

In general, noninvasive brain stimulation techniques lack the capability to target deep brain regions effectively without affecting surrounding areas. As pain encompasses sensory, affective, and cognitive dimensions, effective modulation may require selectively targeting brain regions involved in these processes. One such region is the insula, a key hub for integrating sensori-discriminative and affective-cognitive aspects of pain [65,66,67]. The posterior insula is more strongly associated with processing nociceptive input [68], whereas the anterior insula is most closely associated with affective and evaluative aspects [69]. Dysregulation of insular activity is often observed in chronic pain [66], and lesions in the posterior insula can abolish the unpleasantness of pain while preserving sensory detection [70]. Furthermore, iEEG evidence suggests that nociceptive pain preferentially modulates theta and alpha oscillations in the dorsal posterior insula [71], indicating that this subregion could be a promising stimulation target.

Targeting the insula for pain treatment has been investigated in several studies. A recent rodent study showed that repetitive insular cortex stimulation alleviated neuropathic pain by normalizing synaptic plasticity, specifically reducing phosphorylated CRMP2, AMPAR, and NR2B expression, and dampening insular hyperactivity [72]. A pilot intracranial study in humans showed that anterior insula stimulation raised heat pain thresholds without adverse effects, supporting its role in the affective-cognitive modulation of pain [73]. Together, these converging lines of evidence underscore the insula as a promising therapeutic hub for targeting both sensory and affective dimensions of chronic pain.

However, invasive insular stimulation for pain remains limited in scope, and its clinical application is constrained by surgical risks and outcome variability. A noninvasive method capable of selectively modulating the insula without affecting the surrounding cortex would therefore offer a safer and more accessible therapeutic option while also enabling deeper investigation into the insula’s mechanistic role in pain. The same principle applies to other regions important in pain construction.

**Figure 2:**
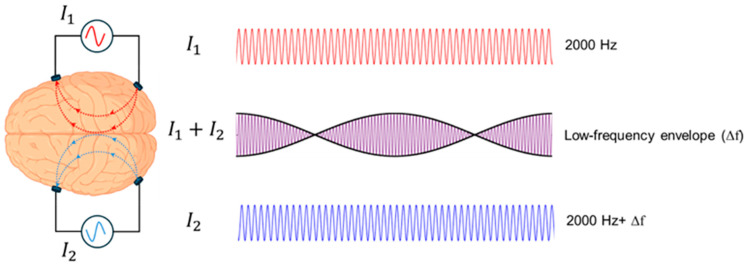
TIS applies two high-frequency alternating currents with slightly different frequencies. Although each carrier frequency is too high to elicit neural firing, their interaction generates a low-frequency envelope capable of modulating neural activity selectively at a targeted focus, minimizing effects on adjacent or superficial regions and reaching deeper targets compared to conventional non-invasive brain stimulation. The red and blue lines represent the two high-frequency stimulation currents, and the purple line represents their interference pattern, with the black curve indicating the resulting low-frequency envelope.

Temporal interference stimulation (TIS) builds on the principle of amplitude-modulated tACS, where two electrode pairs deliver high-frequency currents with slightly different frequencies, creating a low-frequency envelope at their intersections that can modulate neural activity (Figure 2). Grossman et al. demonstrated in mice that such deep activation is possible without engaging in the overlying cortex and that by adjusting current ratios, the stimulation locus can be shifted [74], opening the possibility to selectively target subregions such as specific insular subregions. This steerability and focus make TIS a particularly attractive approach for noninvasively modulating deep structures involved in pain. A systematic review of human TIS studies [75] suggests that although preliminary findings indicate frequency- and target-specific modulation of cortical and subcortical activity, the evidence remains limited by small sample sizes and methodological heterogeneity. Larger, well-controlled trials are needed to establish clinical efficacy.

### 4.6. Recommendations for Future Studies

Taken together, inconsistent findings likely arise from a combination of heterogeneous stimulation parameters, variable sham procedures, insufficient exploration of dose–response and duration effects, limited verification of neural target engagement, diverse pain models, and substantial inter-individual differences. Future research would benefit from systematic testing of stimulation duration and intensity, clearer reporting of modeled intracranial electric fields, standardized sham protocols, larger and adequately powered samples, and direct measures of neural engagement during or immediately after stimulation. Incorporating individualized oscillatory biomarkers, anatomical modeling, and repeated session designs will also help clarify the conditions under which tACS reliably influences pain networks. Addressing these methodological challenges in a coordinated, evidence-based manner is essential to determine whether tACS can provide consistent and meaningful pain modulation. Emerging tACS techniques are increasingly designed to target complex oscillatory dynamics and interactions [76]. Beyond the approaches discussed in this study, other strategies may help advance our understanding of pain and the role of oscillations in its processing. One example is cross-frequency coupled tACS (CFC-tACS) [77], which, rather than applying a single frequency, targets coupled oscillations to modulate their interactions. Another example is broadband tACS, which uses non-sinusoidal patterns that deliver a range of frequencies instead of a narrow sinusoid, thereby mimicking the spectral complexity of natural neural activity. Applying these techniques to pain requires systematic investigation to evaluate their potential contributions to pain modulation mechanisms.

## 5. Conclusions

Across experimental and clinical studies, tACS can modulate oscillatory activity within pain-related networks, but its behavioral effects on pain perception remain variable. Evidence suggests that stimulation outcomes depend on the interaction between stimulation parameters, individual neurophysiology, and contextual brain state. Studies using longer and more focal stimulation, individualized frequency alignment, verification of neural target engagement, and integration with behavioral or cognitive interventions such as expectancy training or pain distraction tend to show more reliable neural and, in some cases, analgesic effects. Progress toward clinical translation will require adequately powered and preregistered studies that combine electric-field modeling with real-time verification of target engagement. Integrating phase- and state-dependent stimulation strategies, including adaptive or closed-loop approaches, represents a critical next step toward precision neuromodulation for pain.

## Figures and Tables

**Table 1:** Summary of key characteristics of tACS studies on pain.

Study	Participant Characteristics	Study Design	Stimulation Montage and Site	tACS Parameters	Control	Outcomes	Main Findings
Experimental Pain tACS studies							
Arendsen et al., 2018 [29]	23 healthy, right-handed (22 female; mean age = 21.4 ± 4.7)	Within-subject, randomized crossover study	Bilateral SM1 (CP3/CP4, 5 × 5 cm pads)	10 Hz, 1 mA pp, ~15–20 min (applied during pressure pain task)	Sham (tRNS 30 s ramp-up/hold/down)	Pain intensity and unpleasantness ratings (NRS) during pressure pain to the middle finger. Participants also performed a visual cue-pain task, where cues predicted the timing of pain stimuli.	Decreased pain intensity and unpleasantness during active tACS vs. sham, only when impending pain intensity was uncertain; no correlations with fear of pain or catastrophizing.
Fathi et al., 2025 [28]	38 healthy participants (19 female; mean age = 28 ± 8.5)	within-subject, randomized crossover, double-blind	Contralateral SM (4 × 1 montage centered on C3/C4)	Individual peak alpha frequency, 2 mA pp, 20 min	Sham (1 min with ramp-up/down)	Heat pain thresholds and ratings + EEG	n.s.; trend toward reduced sensitization in women with active tACS; correlation was observed between SS-PAF and HPT during sham condition.
Ikarashi et al., 2024 [30]	56 healthy, right-handed (27 female; mean age = 21.2 ± 1.0)	Between-subject, randomized, single-blind	Left DLPFC (F3–Fpz, 3 × 3 cm electrodes)	6 Hz (θ) or 20 Hz (β), 1 mA, 20 min	Sham (1 min with ramp-up/down)	Heat pain threshold (HPT) and tolerance (HPTT) using Peltier thermode (right forearm, 35 °C baseline, +0.7 °C/s); repeated measures before, during, and after stimulation;	Pain reduction vs. sham; θ- and β-tACS ↑ HPT during and after stimulation, no effect on HPTT; θ-tACS pain reduction followed an inverted U-shaped relation with simulated E-field in posterior DLPFC, suggesting optimal field strength for maximal pain relief
Li et al., 2025 [31]	80 healthy participants (41 female; mean age = 21.8 ± 2.12)	Between-subjects, double-blinded	Right SM (4 × 1 montage centered on C4 and the 4 surrounding return electrodes were placed at FC2, FC6, CP6, and CP2)	10 Hz, 20 min, 1 mA	Sham (1 min with ramp-up/down)	Ratings of pain intensity and unpleasantness in response to noxious laser stimulation and visual cues manipulated certainty + EEG	n.s.: α-tACS produced no significant pain reduction effect on pain ratings. α-tACS disrupted normal LEP habituation, especially under certain pain expectation, with effects lasting ~30 min. It increased contralateral α_1_ and midfrontal θ activity during anticipation; θ changes persisted. Mediation suggested opposing α-driven analgesic and θ-driven hyperalgesic pathways, with the latter dominating, resulting in no pain reduction.
May et al., 2021 [32]	29 healthy, right-handed (13 female; mean age = 25.7 ± 4.0)	Within-subject, randomized crossover, double-blinded	PFC (F3/F4) or S1 (CP3/CP4), large pads 5 × 5 cm	10 Hz (α) or 80 Hz (γ), 10 min, 1 mA pp	Sham (30 s 10 Hz with ramps)	Tonic heat pain (VAS, continuous ratings), autonomic measures (skin conductance, ECG), EEG (pre/post, 5 min)	n.s. for all conditions; no modulation of pain, autonomic responses, or oscillations. Bayesian analysis supported null, except α-S1 (inconclusive).
Peng et al., 2023 [33]	53 healthy participants (26 active, 27 sham; 32 female; mean = age~21)	Between-subject, double-blinded	Left or right SM1 (C3/C4-centered 4 × 1 HD montage)	10 Hz, 20 min, 1 mA peak-to-peak	Sham (1 min with ramp-up/down)	fMRI during noxious laser stimulation (pain-evoked activity, connectivity), pain ratings (intensity, unpleasantness, NRS)	n.s.; Active α-tACS attenuated pain-evoked activity in bilateral SM1 and left M1 vs. sham. Mediation: reduced SM1 activity indirectly decreased pain ratings. Functional connectivity between SM1 and DLPFC, S1, MCC, and SMA decreased with active vs. sham.
Qi et al., 2025 [34]	31 healthy participants (14 female; mean age = 23 ± 2.3; final after artifact exclusion)	Within-subject, randomized crossover, double-blinded	Contralateral SM1 (4 × 1 HD montage centered on C4)	10 Hz, 30 min, 1.5 mA pp	Sham (1 min with ramp-up/down)	Capsaicin-induced pain (NRS), rest EEG power	Pain reduction vs. sham. Resting α and pain-related θ changes at C4 predicted efficacy (α → θ → pain relief).
Sun et al., 2025 [35]	45 healthy (28 female; mean age = 21 ± 2.4)	Within-subject, randomized crossover, single-blinded	ipsilateral DLPFC (F3-centered, 3 × 1 HD montage)	10 Hz, 30 min, 1.5 mA pp	Sham (1 min with ramp-up/down)	Capsaicin-induced pain (NRS), rest EEG power	Pain reduction vs. sham; Delayed increase in low γ at DLPFC; weak correlation with pain reduction.
Takeuchi & Terui, 2025 [36]	32 healthy participants, 16 pairs (18 females, 14 males; mean age = 21.0 ± 1.4)	Within-subject, randomized crossover	Right S1 (2 cm posterior to C4, concentric 1 × 1 tACS)	10 Hz, peak-to-peak 3 mA, 5 min	Sham (1 min with 30 s ramp-up/down)	Electrical pain intensity ratings (NRS) and empathy scores from the IRI. Participant pairs alternated as pain-receiver and touch-giver while receiving mild electrical pain and gentle brush stroking on the forearm to examine the effects of tACS on touch-induced analgesia.	Though overall pain ratings did not differ across conditions, synchronous dual-brain “hyper”-tACS enhanced social touch-induced pain reduction in low-empathy receivers vs. sham.
Chronic Pain tACS Studies							
Ahn et al., 2019 [37]	20 patients with chronic low back pain (8 male, 12 female; mean pain duration ≈ 85 months).	Within-subject, randomized crossover, double-blind, sham-controlled	Bifrontal (F3/F4, 5 × 5 cm; return 5 × 7 cm at Pz)	10 Hz, 1 mA per F3/F4 (2 mA return at Pz), 40 min	Sham (1 min with ramp-up/down)	Chronic low back pain ratings (DVPRS, ODI), rest EEG (α-power pre/post, correlation with pain relief)	Pain reduction vs. sham; 10 Hz -tACS increased somatosensory α-power, and α enhancement correlated with ↓ chronic pain ratings (DVPRS) and perceived disability (ODI); exploratory Wilcoxon test showed significant ↓ DVPRS, ODI change n.s.
Antal et al. (2020) [38]	25 migraine patients (Active tACS: N = 16, age = 31.1 ± 8.9; Sham: N = 9, age = 28.1 ± 10.5)	Between-subject, randomized parallel groups, double-blinded	Occipital cortex (Oz, 4 × 4 cm) with return at Cz (5 × 7 cm)	140 Hz, 0.4 mA, 15 min per session up to 5 sessions total over a six-week period. Patients were instructed to use tACS during onset of migraine attacks.	Sham (simulator turned off after 30 s)	patients recorded termination of migraine attacks within 2 h post-stimulation (defined as NAS < 1), NAS pain intensity (0–10) before/after migraine attacks, analgesic medication use, and attack recurrence.	During migraines without use of analgesic medication, a significantly greater number of migraine attacks terminated following active tACS compared to sham. Significant ↓ NAS pain severity for active tACS compared to sham at 2–4 h post-stimulation.
Bernardi et al. (2021) [39]	15 individuals diagnosed with fibromyalgia syndrome	Within-subject, randomized crossover, double blind 10 sessions (30 min/day, 5 days/week × 2 weeks) + physiotherapy (60 min/session) with 4-week washout between conditions	Anode positioned individually over scalp region with greatest EEG abnormality (e.g., F3, C3, CP5, C4/Cp2, or similar); cathode over ipsilateral mastoid. large sponge pads (5 × 7 cm)	1–2 mA alternating current Participants with increased slow rhythms (theta, delta, α_1_) received β-tACS at 30 Hz Participants with increased fast rhythms (β, α_2_) received θ-tACS at 4 Hz	Random noise stimulation with random amplitude and frequency, respectively, in the intervals (1–2) mA and (0–100) Hz	Resting EEG (α_1_, θ, δ, β power); Visual Analog Scale (VAS) for pain; neuropsychological and self-report assessments (MoCA, TMT-A/B, HVLT-R, Rey Complex Figure, PROCOG-P, EMQ-R, BDI-II, BSI, STAI); SF-36 for health status.	Pain reduction vs. sham; tailored tACS (4 Hz or 30 Hz, 1–2 mA) increased resting α_1_ power and reduced pain (VAS ↓ at T1) vs. random noise stimulation (RNS). Improved cognitive and neuropsychological performance (MoCA, TMT-B, PROCOG-P, EMQ-R) and reduced depressive symptoms (BDI-II, BSI). EEG normalization mainly over sensorimotor/M1 areas; pain relief effects faded by 4 weeks
Lin et al., 2022 [40]	38 patients with fibromyalgia (30F; mean age 48.6 ± 12.9); 35 completed (active = 18, sham = 17)	Between-subject, randomized, double-blind, sham-controlled	Left M1 (anode = C3; 4 cathodes = Cz, F3, T7, P3; radius ≈ 7.5 cm, 4 × 1 HD montage)	50 Hz, 20 min/session, 10 sessions over 2 weeks	Sham (10 s ramp-up, 19 min 40 s no current, 10 s ramp-down)	NRS (pain intensity), FIQ (quality of life), BAI (anxiety), BDI-II (depression), PSQI (sleep quality), PPT (pressure pain threshold), plasma Tau and Aβ_1–42_	Both groups showed within-group symptom improvement; no significant differences between active and sham in NRS, FIQ, or secondary measures. Active HD-tACS significantly reduced FIQ vs. baseline but not vs. sham. One suicide attempt occurred (likely unrelated). HD-tACS was otherwise well tolerated.
Prim et al., 2019 [41]	20 patients with chronic low back pain (8M, 12F; mean age 43 ± 13; mean pain duration ≈ 7 years)	Within-subject, randomized, crossover, double-blind, sham-controlled	Bilateral prefrontal cortex (F3/F4, 5 × 5 cm) with return at Pz (5 × 7 cm)	10 Hz, 1 mA (zero-to-peak), 40 min; in-phase stimulation; ≥7 days washout	Sham (1 min 10 Hz with ramp-up/down)	ECG for HRV (RSA, LF, LF/HF, SDNN, RMSSD); pain (DVPRS), disability (ODI), and pressure pain threshold (PPT) pre/post	No significant change in RSA or LF/HF. SDNN increased after 10 Hz-tACS vs. sham, indicating modulation of overall autonomic balance. Pain ratings and HRV changes were uncorrelated. Twice as many participants were clinical responders (≥2 pt pain reduction) in active vs. sham, though not statistically significant. Blinding was successful and no adverse effects were reported.

Abbreviations: SM1, primary sensorimotor cortex; S1, primary somatosensory cortex; DLPFC, dorsolateral prefrontal cortex; PFC, prefrontal cortex; ECG, electrocardiogram; EEG, electroencephalography; fMRI, functional magnetic resonance imaging; NRS, numerical rating scale; VAS, visual analog scale; NAS, numerical analog scale; HPT, heat pain threshold; HPTT, heat pain tolerance threshold; PAF, Somatosensory individual peak alpha frequency; SS-PAF, individual peak alpha frequency; α, alpha band; θ, theta band; γ, gamma band; HD, high-definition; pp, peak-to-peak; DVPRS, Defense and Veterans Pain Rating Scale; ODI, Oswestry Disability Index; FIQ, Fibromyalgia Impact Questionnaire; MoCA, Montréal Cognitive Assessment; BSI, Brief Symptom Inventory; SF-36, Short-Form Health Survey; STAI, State–Trait Anxiety Inventory; TMT, Trail Making Test; HVLT-R, Hopkins Verbal Learning Test—Revised; IRI, Interpersonal Reactivity Index; PROCOG-P, Patient-Reported Outcomes in Cognitive Impairment; EMQ-R, Everyday Memory Questionnaire Revised; BAI, Beck Anxiety Inventory; BDI-II, Beck Depression Inventory II; PSQI, Pittsburgh Sleep Quality Index; PPT, pressure pain threshold; HRV, heart rate variability; RSA, respiratory sinus arrhythmia; LF/HF, low-frequency to high-frequency ratio; SDNN, standard deviation of normal-to-normal intervals; RMSSD, root mean square of successive differences; LEP, laser-evoked potential; MCC, midcingulate cortex; SMA, supplementary motor area; n.s., not significant.

## Data Availability

No new data were created or analyzed in this study.

## References

[1] Ploner M., Sorg C., Gross J. (2017). Brain Rhythms of Pain. Trends Cogn. Sci..

[2] Fries P. (2015). Rhythms for Cognition: Communication through Coherence. Neuron.

[3] Foxe J.J., Snyder A.C. (2011). The Role of Alpha-Band Brain Oscillations as a Sensory Suppression Mechanism during Selective Attention. Front. Psychol..

[4] Jensen O., Mazaheri A. (2010). Shaping Functional Architecture by Oscillatory Alpha Activity: Gating by Inhibition. Front. Hum. Neurosci..

[5] Nir R.R., Sinai A., Moont R., Harari E., Yarnitsky D. (2012). Tonic Pain and Continuous EEG: Prediction of Subjective Pain Perception by Alpha-1 Power during Stimulation and at Rest. Clin. Neurophysiol..

[6] May E.S., Butz M., Kahlbrock N., Hoogenboom N., Brenner M., Schnitzler A. (2012). Pre- and Post-Stimulus Alpha Activity Shows Differential Modulation with Spatial Attention during the Processing of Pain. Neuroimage.

[7] Huneke N.T.M., Brown C.A., Burford E., Watson A., Trujillo-Barreto N.J., El-Deredy W., Jones A.K.P. (2013). Experimental Placebo Analgesia Changes Resting-State Alpha Oscillations. PLoS ONE.

[8] Walton K.D., Dubois M., Llinás R.R. (2010). Abnormal Thalamocortical Activity in Patients with Complex Regional Pain Syndrome (CRPS) Type I. Pain.

[9] Walton K.D., Llinás R.R., Kruger L., Light A.R. (2010). Central Pain as a Thalamocortical Dysrhythmia: A Thalamic Efference Disconnection?. Translational Pain Research: From Mouse to Man.

[10] Sarnthein J., Stern J., Aufenberg C., Rousson V., Jeanmonod D. (2006). Increased EEG Power and Slowed Dominant Frequency in Patients with Neurogenic Pain. Brain.

[11] Stern J., Jeanmonod D., Sarnthein J. (2006). Persistent EEG Overactivation in the Cortical Pain Matrix of Neurogenic Pain Patients. Neuroimage.

[12] Furman A.J., Thapa T., Summers S.J., Cavaleri R., Fogarty J.S., Steiner G.Z., Schabrun S.M., Seminowicz D.A. (2019). Cerebral Peak Alpha Frequency Reflects Average Pain Severity in a Human Model of Sustained, Musculoskeletal Pain. J. Neurophysiol..

[13] de Vries M., Wilder-Smith O.H.G., Jongsma M.L.A., van den Broeke E.N., Arns M., van Goor H., van Rijn C.M. (2013). Altered Resting State EEG in Chronic Pancreatitis Patients: Toward a Marker for Chronic Pain. J. Pain Res..

[14] Furman A.J., Meeker T.J., Rietschel J.C., Yoo S., Muthulingam J., Prokhorenko M., Keaser M.L., Goodman R.N., Mazaheri A., Seminowicz D.A. (2018). Cerebral Peak Alpha Frequency Predicts Individual Differences in Pain Sensitivity. Neuroimage.

[15] Huber F.A., Kell P.A., Shadlow J.O., Rhudy J.L. (2025). Cerebral Peak Alpha Frequency: Associations with Chronic Pain Onset and Pain Modulation. Neurobiol. Pain.

[16] May E.S., Tiemann L., Gil Ávila C., Bott F.S., Hohn V.D., Gross J., Ploner M. (2025). Assessing the Predictive Value of Peak Alpha Frequency for the Sensitivity to Pain. Pain.

[17] Bott F.S., Zebhauser P.T., Hohn V.D., Turgut Ö., May E.S., Tiemann L., Gil Ávila C., Heitmann H., Nickel M.M., Day M.A. (2025). Exploring Electroencephalographic Chronic Pain Biomarkers: A Mega-Analysis. EBioMedicine.

[18] Bott F.S., Nickel M.M., Hohn V.D., May E.S., Ávila C.G., Tiemann L., Gross J., Ploner M. (2023). Local Brain Oscillations and Interregional Connectivity Differentially Serve Sensory and Expectation Effects on Pain. Sci. Adv..

[19] Kenefati G., Rockholt M.M., Ok D., McCartin M., Zhang Q., Sun G., Maslinski J., Wang A., Chen B., Voigt E.P. (2023). Changes in Alpha, Theta, and Gamma Oscillations in Distinct Cortical Areas Are Associated with Altered Acute Pain Responses in Chronic Low Back Pain Patients. Front. Neurosci..

[20] Barbosa S.P., Junqueira Y.N., Akamatsu M.A., Marques L.M., Teixeira A., Lobo M., Mahmoud M.H., Omer W.E., Pacheco-Barrios K., Fregni F. (2024). Resting-State Electroencephalography Delta and Theta Bands as Compensatory Oscillations in Chronic Neuropathic Pain: A Secondary Data Analysis. Brain Netw. Modul..

[21] Shirvalkar P., Prosky J., Chin G., Ahmadipour P., Sani O.G., Desai M., Schmitgen A., Dawes H., Shanechi M.M., Starr P.A. (2023). First-in-Human Prediction of Chronic Pain State Using Intracranial Neural Biomarkers. Nat. Neurosci..

[22] Bikson M., Esmaeilpour Z., Adair D., Kronberg G., Tyler W.J., Antal A., Datta A., Sabel B.A., Nitsche M.A., Loo C. (2019). Transcranial Electrical Stimulation Nomenclature. Brain Stimul..

[23] Helfrich R.F., Schneider T.R., Rach S., Trautmann-Lengsfeld S.A., Engel A.K., Herrmann C.S. (2014). Entrainment of Brain Oscillations by Transcranial Alternating Current Stimulation. Curr. Biol..

[24] Ali M.M., Sellers K.K., Fröhlich F. (2013). Transcranial Alternating Current Stimulation Modulates Large-Scale Cortical Network Activity by Network Resonance. J. Neurosci..

[25] Huang W.A., Stitt I.M., Negahbani E., Passey D.J., Ahn S., Davey M., Dannhauer M., Doan T.T., Hoover A.C., Peterchev A.V. (2021). Transcranial Alternating Current Stimulation Entrains Alpha Oscillations by Preferential Phase Synchronization of Fast-Spiking Cortical Neurons to Stimulation Waveform. Nat. Commun..

[26] Vossen A., Gross J., Thut G. (2015). Alpha Power Increase after Transcranial Alternating Current Stimulation at Alpha Frequency (a-TACS) Reflects Plastic Changes Rather than Entrainment. Brain Stimul..

[27] Riddle J., Frohlich F. (2021). Targeting Neural Oscillations with Transcranial Alternating Current Stimulation. Brain Res..

[28] Fathi Y., Dissassuca F., Ricci G., Liberati G. (2025). Individualised Alpha-TACS for Modulating Pain Perception and Neural Oscillations: A Sham-Controlled Study in Healthy Participants. Eur. J. Pain.

[29] Arendsen L.J., Hugh-Jones S., Lloyd D.M. (2018). Transcranial Alternating Current Stimulation at Alpha Frequency Reduces Pain When the Intensity of Pain Is Uncertain. J. Pain.

[30] Ikarashi H., Otsuru N., Gomez-Tames J., Hirata A., Nagasaka K., Miyaguchi S., Sakurai N., Ohno K., Kodama N., Onishi H. (2024). Modulation of Pain Perception through Transcranial Alternating Current Stimulation and Its Nonlinear Relationship with the Simulated Electric Field Magnitude. Eur. J. Pain.

[31] Li X., Jin R., Lu X., Zhan Y., Jiang N., Peng W. (2025). Alpha Transcranial Alternating Current Stimulation Modulates Pain Anticipation and Perception in a Context-Dependent Manner. Pain.

[32] May E.S., Hohn V.D., Nickel M.M., Tiemann L., Gil Ávila C., Heitmann H., Sauseng P., Ploner M. (2021). Modulating Brain Rhythms of Pain Using Transcranial Alternating Current Stimulation (TACS)-a Sham-Controlled Study in Healthy Human Participants. J. Pain.

[33] Peng W., Zhan Y., Jin R., Lou W., Li X. (2023). Aftereffects of Alpha Transcranial Alternating Current Stimulation over the Primary Sensorimotor Cortex on Cortical Processing of Pain. Pain.

[34] Qi X., Jia T., Sun B., Xia J., Wang C.X., Hong Z., Zhang Y., Yang H., Zhang C., Liu J. (2025). Individual Differences in Resting Alpha Band Power and Changes in Theta Band Power during Sustained Pain Are Correlated with the Pain-Relieving Efficacy of Alpha HD-TACS on SM1. Neuroimage.

[35] Sun B., Zhang C., Zhang Q., Xu X., Liu J., Yang H. (2025). Analgesic Aftereffects of Alpha High-Definition Transcranial Alternating Current Stimulation over the DLPFC during Experimental Pain. Neuroimage.

[36] Takeuchi N., Terui Y. (2025). Synchronal Dual Brain Stimulation over the Somatosensory Cortex Modulated Social Touch-Induced Analgesia Depending on Empathy. J. Pain.

[37] Ahn S., Prim J.H., Alexander M.L., McCulloch K.L., Fröhlich F. (2018). Identifying and Engaging Neuronal Oscillations by Transcranial Alternating Current Stimulation in Patients with Chronic Low Back Pain: A Randomized, Crossover, Double-Blind, Sham-Controlled Pilot Study. J. Pain.

[38] Antal A., Bischoff R., Stephani C., Czesnik D., Klinker F., Timäus C., Chaieb L., Paulus W. (2020). Low Intensity, Transcranial, Alternating Current Stimulation Reduces Migraine Attack Burden in a Home Application Set-Up: A Double-Blinded, Randomized Feasibility Study. Brain Sci..

[39] Bernardi L., Bertuccelli M., Formaggio E., Rubega M., Bosco G., Tenconi E., Cattelan M., Masiero S., Felice A. (2020). Del Beyond Physiotherapy and Pharmacological Treatment for Fibromyalgia Syndrome: Tailored TACS as a New Therapeutic Tool. Eur. Arch. Psychiatry Clin. Neurosci..

[40] Lin A.P., Chiu C.C., Chen S.C., Huang Y.J., Lai C.H., Kang J.H. (2022). Using High-Definition Transcranial Alternating Current Stimulation to Treat Patients with Fibromyalgia: A Randomized Double-Blinded Controlled Study. Life.

[41] Prim J.H., Ahn S., Davila M.I., Alexander M.L., McCulloch K.L., Fröhlich F. (2019). Targeting the Autonomic Nervous System Balance in Patients with Chronic Low Back Pain Using Transcranial Alternating Current Stimulation: A Randomized, Crossover, Double-Blind, Placebo-Controlled Pilot Study. J. Pain Res..

[42] Bland N.S., Sale M.V. (2019). Current Challenges: The Ups and Downs of TACS. Exp. Brain Res..

[43] Hashmi J.A., Baliki M.N., Huang L., Baria A.T., Torbey S., Hermann K.M., Schnitzer T.J., Apkarian A.V. (2013). Shape Shifting Pain: Chronification of Back Pain Shifts Brain Representation from Nociceptive to Emotional Circuits. Brain.

[44] Baliki M.N., Petre B., Torbey S., Herrmann K.M., Huang L., Schnitzer T.J., Fields H.L., Apkarian A.V. (2012). Corticostriatal Functional Connectivity Predicts Transition to Chronic Back Pain. Nat. Neurosci..

[45] Vachon-Presseau E., Centeno M.V., Ren W., Berger S.E., Tétreault P., Ghantous M., Baria A., Farmer M., Baliki M.N., Schnitzer T.J. (2016). The Emotional Brain as a Predictor and Amplifier of Chronic Pain. J. Dent. Res..

[46] Baliki M.N., Mansour A.R., Baria A.T., Apkarian A.V. (2014). Functional Reorganization of the Default Mode Network across Chronic Pain Conditions. PLoS ONE.

[47] Kaplan C.M., Kelleher E., Irani A., Schrepf A., Clauw D.J., Harte S.E. (2024). Deciphering Nociplastic Pain: Clinical Features, Risk Factors and Potential Mechanisms. Nat. Rev. Neurol..

[48] Napadow V., LaCount L., Park K., As-Sanie S., Clauw D.J., Harris R.E. (2010). Intrinsic Brain Connectivity in Fibromyalgia Is Associated with Chronic Pain Intensity. Arthritis Rheum..

[49] Kuner R., Flor H. (2016). Structural Plasticity and Reorganisation in Chronic Pain. Nat. Rev. Neurosci..

[50] Ellingsen D.M., Beissner F., Moher Alsady T., Lazaridou A., Paschali M., Berry M., Isaro L., Grahl A., Lee J., Wasan A.D. (2021). A Picture Is Worth a Thousand Words: Linking Fibromyalgia Pain Widespreadness from Digital Pain Drawings with Pain Catastrophizing and Brain Cross-Network Connectivity. Pain.

[51] Babiloni C., Brancucci A., Del Percio C., Capotosto P., Arendt-Nielsen L., Chen A.C.N., Rossini P.M. (2006). Anticipatory Electroencephalography Alpha Rhythm Predicts Subjective Perception of Pain Intensity. J. Pain.

[52] Tu Y., Zhang Z., Tan A., Peng W., Hung Y.S., Moayedi M., Iannetti G.D., Hu L. (2016). Alpha and Gamma Oscillation Amplitudes Synergistically Predict the Perception of Forthcoming Nociceptive Stimuli. Hum. Brain Mapp..

[53] Chang M.C., Briand M.M., Boudier-Revéret M., Yang S. (2023). Effectiveness of Transcranial Alternating Current Stimulation for Controlling Chronic Pain: A Systematic Review. Front. Neurol..

[54] Haslacher D., Cavallo A., Reber P., Kattein A., Thiele M., Nasr K., Hashemi K., Sokoliuk R., Thut G., Soekadar S.R. (2024). Working Memory Enhancement Using Real-Time Phase-Tuned Transcranial Alternating Current Stimulation. Brain Stimul..

[55] Fassi L., Hochman S., Daskalakis Z.J., Blumberger D.M., Kadosh R.C. (2023). The Importance of Individual Beliefs in Assessing Treatment Efficacy: Insights from Neurostimulation Studies. Elife.

[56] Turner C., Jackson C., Learmonth G. (2021). Is the “End-of-Study Guess” a Valid Measure of Sham Blinding during Transcranial Direct Current Stimulation?. Eur. J. Neurosci..

[57] Dehghani A., Gantz D.M., Murphy E.K., Nitsche M.A., Halter R.J., Wager T.D. (2025). Transcranial Direct Current Stimulation of Primary Motor Cortex Reduces Thermal Pain. Pain.

[58] Witkowski M., Garcia-Cossio E., Chander B.S., Braun C., Birbaumer N., Robinson S.E., Soekadar S.R. (2016). Mapping Entrained Brain Oscillations during Transcranial Alternating Current Stimulation (TACS). Neuroimage.

[59] Haslacher D., Nasr K., Robinson S.E., Braun C., Soekadar S.R. (2021). Stimulation Artifact Source Separation (SASS) for Assessing Electric Brain Oscillations during Transcranial Alternating Current Stimulation (TACS). Neuroimage.

[60] Haslacher D., Narang A., Sokoliuk R., Cavallo A., Reber P., Nasr K., Santarnecchi E., Soekadar S.R. (2023). In Vivo Phase-Dependent Enhancement and Suppression of Human Brain Oscillations by Transcranial Alternating Current Stimulation (TACS). Neuroimage.

[61] Thiele C., Zaehle T., Haghikia A., Ruhnau P. (2021). Amplitude Modulated Transcranial Alternating Current Stimulation (AM-TACS) Efficacy Evaluation via Phosphene Induction. Sci. Rep..

[62] Krause M.R., Vieira P.G., Thivierge J.P., Pack C.C. (2022). Brain Stimulation Competes with Ongoing Oscillations for Control of Spike Timing in the Primate Brain. PLoS Biol..

[63] Fiene M., Schwab B.C., Misselhorn J., Herrmann C.S., Schneider T.R., Engel A.K. (2020). Phase-Specific Manipulation of Rhythmic Brain Activity by Transcranial Alternating Current Stimulation. Brain Stimul..

[64] Schreglmann S.R., Wang D., Peach R.L., Li J., Zhang X., Latorre A., Rhodes E., Panella E., Cassara A.M., Boyden E.S. (2021). Non-Invasive Suppression of Essential Tremor via Phase-Locked Disruption of Its Temporal Coherence. Nat. Commun..

[65] Isnard J., Magnin M., Jung J., Mauguière F., Garcia-Larrea L. (2011). Does the Insula Tell Our Brain That We Are in Pain?. Pain.

[66] McBenedict B., Petrus D., Pires M.P., Pogodina A., Agbor D.B.A., Ahmed Y.A., Ceron J.I.C., Balaji A., Abrahão A., Pessôa B.L. (2024). The Role of the Insula in Chronic Pain and Associated Structural Changes: An Integrative Review. Cureus.

[67] Kurth F., Zilles K., Fox P.T., Laird A.R., Eickhoff S.B. (2010). A Link between the Systems: Functional Differentiation and Integration within the Human Insula Revealed by Meta-Analysis. Brain Struct. Funct..

[68] Brooks J.C.W., Tracey I. (2007). The Insula: A Multidimensional Integration Site for Pain. Pain.

[69] Bastuji H., Frot M., Perchet C., Hagiwara K., Garcia-Larrea L. (2018). Convergence of Sensory and Limbic Noxious Input into the Anterior Insula and the Emergence of Pain from Nociception. Sci. Rep..

[70] Berthier M., Starkstein S., Leiguarda R. (1988). Asymbolia for Pain: A Sensory-Limbic Disconnection Syndrome. Ann. Neurol..

[71] Liberati G., Algoet M., Santos S.F., Ribeiro-Vaz J.G., Raftopoulos C., Mouraux A. (2019). Tonic Thermonociceptive Stimulation Selectively Modulates Ongoing Neural Oscillations in the Human Posterior Insula: Evidence from Intracerebral EEG. Neuroimage.

[72] Kim K., Nan G., Bak H., Kim H.Y., Kim J., Cha M., Lee B.H. (2024). Insular Cortex Stimulation Alleviates Neuropathic Pain through Changes in the Expression of Collapsin Response Mediator Protein 2 Involved in Synaptic Plasticity. Neurobiol. Dis..

[73] Liu C.C., Moosa S., Quigg M., Elias W.J. (2021). Anterior Insula Stimulation Increases Pain Threshold in Humans: A Pilot Study. J. Neurosurg..

[74] Grossman N., Bono D., Dedic N., Kodandaramaiah S.B., Rudenko A., Suk H.-J., Cassara A.M., Neufeld E., Kuster N., Tsai L.-H. (2017). Noninvasive Deep Brain Stimulation via Temporally Interfering Electric Fields. Cell.

[75] Mansourinezhad P., Mestrom R.M.C., Klooster D.C.W., Sprengers M., Boon P.A.J.M., Paulides M.M. (2025). Systematic Review of Experimental Studies in Humans on Transcranial Temporal Interference Stimulation. J. Neural Eng..

[76] Barzegar S., Kakies C.F.M., Ciupercӑ D., Wischnewski M. (2025). Transcranial Alternating Current Stimulation for Investigating Complex Oscillatory Dynamics and Interactions. Int. J. Psychophysiol..

[77] Alekseichuk I., Turi Z., Amador de Lara G., Antal A., Paulus W. (2016). Spatial Working Memory in Humans Depends on Theta and High Gamma Synchronization in the Prefrontal Cortex. Curr. Biol..

